# Effect of Different Compatibilizers on Injection-Molded Green Fiber-Reinforced Polymers Based on Poly(lactic acid)-Maleinized Linseed Oil System and Sheep Wool

**DOI:** 10.3390/polym11091514

**Published:** 2019-09-17

**Authors:** Franciszek Pawlak, Miguel Aldas, Juan López-Martínez, María Dolores Samper

**Affiliations:** 1 Faculty of Technology and Chemical Engineering, University of Science and Technology in Bydgoszcz, Seminaryjna 3, PL-85326 Bydgoszcz, Poland; 2 Instituto de Tecnología de Materiales, Universitat Politècnica de València, Plaza Ferrandiz y Carbonelle, 03801 Alcoy-Alicante, Spain; jlopezm@mcm.upv.es (J.L.-M.); masammad@upvnet.upv.es (M.D.S.); 3 Departamento de Ciencia de Alimentos y Biotecnología, Facultad de Ingeniería Química y Agroindustria, Escuela Politécnica Nacional, Ladrón de Guevera E11-253, Quito 170517, Ecuador

**Keywords:** poly(lactic acid), wool, fiber reinforced polymer (FRP), green materials, coupling agent, silane, alkoxide, compatibilizers

## Abstract

A method to modify polymers is that of introducing fibers in a matrix to produce a fiber-reinforced polymer (FRP). Consequently, the aim of this work was to study the compatibility effect of four coupling agents on wool FRP properties, using poly(lactic acid) plasticized with maleinized linseed oil as polymer matrix. The content of wool assessed was 1 phr. The compatibilizers were (3-(2-aminoethylamino)propyl)-trimethoxysilane, trimethoxy (2-(7-oxabicyclo (4.1.0)hept-3-yl) ethyl) silane, tris(2-methoxyethoxy)(vinyl) silane and titanium (IV) (triethanolaminate)isopropoxide. Initially, wool was modified with coupling agents in an acetone/water (50/50) solution. Mechanical properties were evaluated by tensile and flexural properties, hardness by Shore D measurement and impact resistance by Charpy’s energy. Differential scanning calorimetry, dynamic thermo-mechanical analysis, and thermogravimetric analysis were conducted to evaluate the interaction among components and the effect of the coupling agents on the thermal properties of the original material. Color, wettability and scanning electron microscopy were used to describe physical and microstructural properties. Modification of fibers allows achieving improved mechanical properties and changes the thermal properties of the FRPs slightly. Coupling agent treatment helps to formulate PLA–MLO and sheep wool materials and to improve their performance, thereby creating a broader spectrum of applications for PLA maintaining the bio-based character of the material.

## 1. Introduction

Nowadays, an increased interest can be observed for biodegradable and bio-based materials as an alternative to petroleum-based polymers. Implementation of biomaterials for short-term and disposable applications is crucial for material utilization, as the continuous development of bio-economy in material science might decrease petroleum-based material dependence [1,2,3]. Due to a short life cycle and their renewability, such materials are more and more often considered for industrial application. In fact, the use of bio-based materials could contribute to solving many environmental problems such as the depletion of non-renewable resources or environmental pollution [4,5,6]. A growing demand for bioplastic materials is caused by a reduction in usage of non-renewable materials, in order to prevent the accumulation of plastic waste in the future [5,7]. Biopolymers’ variety and multiple applications are also increasing society’s awareness of materials that derive from renewable resources, minimizing the environmental impact. Most common biopolymers used for industrial applications are poly(lactic acid)-PLA, poly(hydroxybutyrate)-PHB and thermoplastic starch-TPS, which represent conventional polymers whose monomers are obtained from agro-resources [8,9]. The demand for biopolymers is frequently caused by the biodegradable character of such materials which allows them to be recycled using an environmentally friendly composting method [1]. Such plastic material can be easily disintegrated in compost conditions resulting in changes from bioplastic into water, carbon dioxide, and hummus, by the action of microorganisms [8,10]. Moreover, the eco-friendly character of biopolymers encourages further modification of material with natural origin additives that allows the material properties to be improved and maintains its biodegradable character [11,12].

Modern construction materials are more often manufactured by reinforcement in order to create new materials with tailored properties for a specific application. One of the most promising strategies for materials improvement is creating natural fiber-reinforced polymer (FRP) which allows modification of a polymer matrix with bio-based industrial by-product waste as a replacement for conventional inorganic additives. Bio-additives are promising for manufacturing due to their biocompatibility, sustainability and surface group functionality [13,14,15]. Although modification with renewable fibers can be profitable for polymer composites or FRP (i.e., by decreasing cost of production), higher filler rate or size often decrease mechanical properties due to debonding effect of the filler from a polymer matrix, and reduces possible fields of application [11,15]. Therefore, improving the mechanical properties of the FRP is crucial for material applications. In consequence, there are mainly two strategies to improve stress transfer between a polymer matrix and filler: i) surface treatment of filler; or ii) reducing force and viscosity in the system by plasticization. [5,11,16,17,18].

Poly(lactic acid) (PLA) is one of the most promising biopolymers due to its balanced mechanical properties, easy processing, and availability in the market [2,5,8]. This biopolymer can be synthesized from simple sugars derived from biomass, such as corn, sugar beets or rice, to lactic acid [11,19]. This bio-based polymer has currently the highest potential for replacing petroleum-based synthetic polymers. PLA has comparable mechanical and thermal properties to such polymers as polystyrene or polyethylene terephthalate and additionally its biodegradable and biocompatible character makes it suitable even for food packaging or medical applications [4,12,16,19]. Although the material presents such advantages as high transparency, resistance to moisture and fats, and natural origin, some of the properties such as low flexibility or elongation at break or low crystallization rate should be enhanced for a broader spectrum of industrial applications [2,5,16]. Improvement of material properties can be obtained through many approaches such as plasticization, polymer blending or incorporation of nanofillers [8,20]. The variety of advantages and disadvantages of PLA–based materials currently makes it suitable mainly for short-term and disposable application [2].

On the other hand, vegetable oils are currently desired in the industry because of their chemical structure and bio-origin [6,21]. Linseed oil is one of the most common vegetable oils for coating production due to its chemical structure containing a few carbon–carbon double bonds per fatty acid. High reactivity allows a high degree of oil functionalization and provides better plasticizing effect between a polymer matrix and the oil, as unmodified vegetable oils are incompatible with most polymers [6,9,17,19]. Modification of linseed oil within maleinization or epoxidation processes allows the oil to interact better with the PLA matrix, which provides a plasticization effect and allows crystallization to occur at lower energy. In such cases, maleinized linseed oil (MLO) can contribute to making the material more flexible due to the increase in chain mobility and compatibility due to easier crystallite build-up [8,10,17,19,20]. In addition, the positive effect of MLO and similar chemically modified vegetable oils (epoxidized linseed oil (ELO) and epoxidized soybean oil (ESO)) as plasticizers was studied for various polymer mixtures such as PLA with MLO [19], poly(butylene succinate) (PBS) with MLO [22] and polyvinyl chloride (PVC) with ELO [23]. 

Finally, wool is an example of high-quality natural fibers consisting of proteins, which are highly suitable for textile applications. The textile industry requires a specific type of wool, such as the Merino sheep wool, which generates plenty of wool as post-consumer waste. In addition, the Merino breed holds the quasi-monopoly of textile applications for sheep’s wool thanks to its great softness [24,25]. However, there is a breed of sheep that are specifically raised for their milk and to produce cheese—wool derived from this industry is not suitable for the textile sector. In consequence, the wool coming from this activity is considered a by-product [26,27,28,29,30]. Additionally, low material cost, biodegradability and the low energy required for processing make natural fibers a good replacement for conventional synthetic fibers such as carbon or glass fibers, from both an economic and environmental point of view. However, bio-origin fibers often present significantly lower mechanical properties or higher water uptake compared to synthetic fibers, which can be enhanced by fiber surface modification [5,17,29,31,32]. The hydrophilic surface of wool fibers needs to be chemically modified in order to improve compatibility with a hydrophobic polymer matrix. One of the most common methods to change the interaction of natural fibers with water and to improve the polymer–fiber interaction is treatment with silanes. In the presence of moisture, silanes form strong chemical bonding with fiber and are able to bond with the polymer matrix. Such formulation improves matrix adhesion and stabilizes material properties. The silane coupling effect with a polymer matrix has been observed for both inorganic and natural fibers. In the case of wool, the surface of the fiber is covered with a coupling agent, which has a role to increase the interaction with polymer chains. Such increase of interaction between polymer matrixes was also observed for cellulose fibers or mineral fibers [6,32,33].

In consequence, in this work four different coupling agents were employed as wool fiber compatibilizer in FRP based on PLA plasticized with MLO, to study their compatibility effect on the material. The FRPs were processed by extrusion–compounding and injection molding and mechanical, thermal, physical and microstructural properties were assessed.

## 2. Materials and Methods 

### 2.1. Materials

Poly(lactic acid)-PLA Ingeo Biopolymer 6201D was supplied by NatureWorks LLC (Minnetonka, Minnesota, USA) and was used as the polymer matrix for the FRP materials. The commercial grade contains 2% D-lactic acid; the material is characterized by a density of 1.24 g/cm^3^ and a melt flow index (MFI) in the 15–30 g/(10 min) range measured at 210 °C. Its molecular weight is about 70,000 g/mol [34]. Plasticizer used in the preparation was a maleinized linseed oil (MLO) Veomer Lin. It was supplied by Vendeputte (Mouscron, Belgium) with a viscosity of 10 dPa s at 20 °C and an acid value of 105–130 mg KOH/g. Wool was obtained in the form of raw material from Basque Country (Basque Country, Spain), which was previously cleaned and prepared by following the multistep procedure. The animal-derived fibers were cut and cleaned from dirt by a washed solution containing 0.2 wt % of sodium carbonate and 2 wt % of commercial detergent. As prepared wool, fibers were carded to orientate fibers in one direction and were cut into the length of 1 cm, achieving fibers with a length to diameter (L/D) ratio of approximately 200.

**Figure 1:**
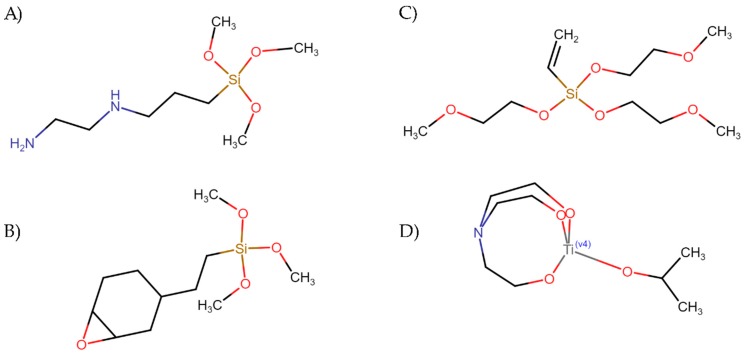
Chemical structure of coupling agents: (**A**) (3-(2-aminoethylamino)propyl)-trimethoxysilane; (**B**) trimethoxy (2-(7-oxabicyclo(4.1.0)hept-3-yl)ethyl]silane; (**C**) tris(2-methoxyethoxy)(vinyl) silane; and (**D**) titanium (IV) (triethanolaminate)isopropoxide.

Surface modification was carried out with the following coupling agents provided by Sigma-Aldrich (Schnelldorf, Germany): (3-(2-aminoethylamino)propyl)-trimethoxysilane (labeled as coupling agent A), trimethoxy (2-(7-oxabicyclo(4.1.0)hept-3-yl)ethyl)silane (labeled as coupling agent B), tris(2-methoxyethoxy)(vinyl) silane (labeled as coupling agent C) and titanium (IV) (triethanolaminate)isopropoxide (labeled as coupling agent D). Silanes and alkoxide were used for improving the load transfer between the polymer matrix and fiber leading to better mechanical performance, as this is beneficial for inorganic fibers [6]. The chemical structure of all coupling agents is presented in Figure 1.

### 2.2. Surface Treatment of Wool Fibers

**Figure 2:**
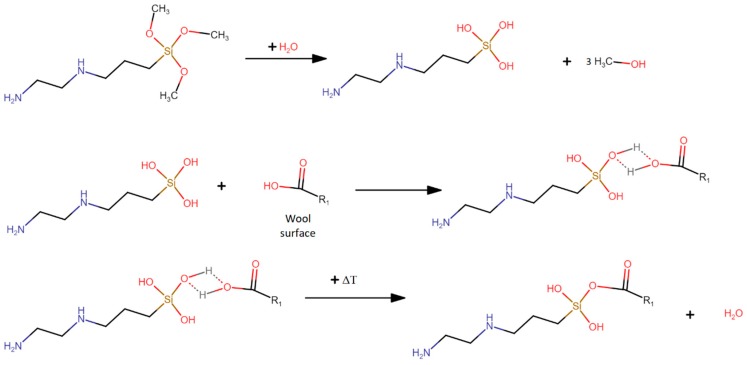
Scheme of coupling reaction of wool with coupling agent (3-(2-aminoethylamino)propyl)-trimethoxysilane.

The study was performed on five types of wool: the unmodified wool, and the wool modified with 1 phr of coupling agents A, B, C and D, respectively. For modifications, 1 phr of coupling agent was added to a water/acetone solution (50:50 wt %) and was mixed for 30 min. It is preferable to use this solution because the presence of ethanol can result in desorption of coupling agent from surface and acetone is then inert for the reaction but dissolves coupling agent [35]. Subsequently, 100 phr of wool was immersed in the solution, which was mixed for 1 h at room temperature. Once prepared, modified wool was dried at 50 °C for 48 h until complete evaporation of solvents. An example of the chemical reaction scheme for fiber modification is shown in Figure 2.

### 2.3. Characterization of Wool Fiber Treatment

To verify the treatment of the wool, attenuated total reflectance Fourier transform infrared spectroscopy (FTIR) and thermogravimetric analyses (TGA) were performed in unmodified wool (UW) and in all the wool fibers modified with coupling agents A, B, C and D (labeled as WA, WB, WC and WD, respectively). FTIR was recorded using a Perkin Elmer Spectrum BX FTIR system (Beaconsfield, United Kingdom) within the range of 4000–650 cm^−1^. The resolution used was 16 cm^−1^ and 20 scans were fulfilled. TGA was conducted in Linseis TGA PT1000 (Selb, Germany) in a dynamic mode with a heating rate of 10 °C/min, from 30 to 700 °C, in a nitrogen atmosphere (flow 20 cm^3^/min). The onset degradation temperatures (*T*_5_) were determined at 5% of mass loss, while temperatures of the maximum decomposition rate (*T*_max_) were calculated in the temperature of the peak from the first derivative of the TGA curves (DTG). TGA analyses were performed by triplicate and the average and standard deviation of the values are reported. Significance in the data differences was statistically analyzed with OriginPro 8 software (OriginLab, Northampton, MA, USA). The significant differences among the wool fiber samples were recorded at 95% confidence level according to Tukey’s test using the one-way analysis of variance (ANOVA). 

### 2.4. Fiber Reinforce Polymer Manufacturing

Wool fibers and MLO were weighed in 1 phr and 10 phr rate content, respectively, based on 100 parts of PLA pellet. For the study, six kinds of material were prepared: PLA containing MLO (labeled as PLA–MLO), PLA with MLO with unmodified wool (PLA–MLO–UW) and PLA with MLO, plus treated wool with the four different coupling agents: (3-(2-aminoethylamino)propyl)-trimethoxysilane (labeled as PLA–MLO–WA), trimethoxy (2-(7-oxabicyclo(4.1.0)hept-3-yl)ethyl)silane (labeled as PLA–MLO–WB), tris(2-methoxyethoxy)(vinyl) silane (labeled as PLA–MLO–WC) and titanium (IV) (triethanolaminate)isopropoxide (labeled as PLA–MLO–WD). As prepared, the components of the formulations were dried and then pre-mixed in a plastic container. Afterward, each formulation was extruded in a co-rotating twin-screw extruder, L/D ratio of 25 from Dupra S.L (Castalla, Spain), in a temperature profile of 150-170-180-185 °C (from hopper to die), with a rotation speed 20 rpm. The material was ground after the extrusion in mill manufactured by Silmisa (Onil, Spain), at 500 rpm. The obtained pellets size was between 3.5 and 4 mm. Later, the pellets were dried in an oven for 24 h in 50 °C and then it was processed by injection molding to obtain test specimens, in an injection molding machine (Sprinter-11, Erinca S,L., Barcelona, Spain) at a temperature profile from 160-170-180-180 °C (from hopper to die). The injected test samples were used for further characterization of the FRP material.

### 2.5. Characterization of the FRP

Tensile and flexural tests were performed according to standard tests methods ISO 527 and ISO 178, respectively. Both tests were performed in a universal test machine Ibertest ELIB-50-W (Madrid, Spain) with a load cell of 5 kN and a crosshead rate of 10 mm/min. Charpy’s impact resistance was determined in an equipment manufactured by Metrotec (San Sebastian, Spain) with a pendulum of 6 J. Hardness test was performed in Shore D durometer, model 673-D manufactured by Instruments J. Bot S.A. (Barcelona, Spain). Five specimens were characterized for each test and the mean and standard deviation of the values are reported. Additionally, the toughness of each formulated material was determined. The values were obtained calculating the area under the stress–strain curves of the tensile test. The area was calculated using the OriginPro2015 program [36]. Significance in the mechanical data differences was statistically analyzed under the same conditions specified for TGA data treatment; that is, using the one-way analysis of variance (ANOVA), by means of OriginPro 8 software, at 95% confidence level according to Tukey’s test for the significant differences among formulations.

Thermogravimetric analyses (TGA) were performed in Linseis TGA PT1000 (Selb, Germany) in a dynamic mode with a heating rate of 10 °C/min, from 30 to 700 °C, in a nitrogen atmosphere (flow 20 cm^3^/min). The onset degradation temperatures (*T*_5_) were determined at 5% of mass loss, while temperatures of the maximum decomposition rate (*T*_max_) were calculated in the temperature of the peak from the first derivative of the TGA curves (DTG). The mass losses at 300 °C and 350 °C are reported as well. 

Differential scanning calorimetry (DSC) was carried out in a Mettler DSC821e (Toledo, Spain) with a thermal cycle of heating from 30 to 180 °C, then cooling from 180 to 30 °C and a second heating from 30 to 220 °C, with a heating rate for all cycles of 10 °C/min and under a nitrogen atmosphere (flow 20 cm^3^/min). Thermal properties were evaluated based on the second heating of the DSC curve. Glass transition temperature (*T*_g_), melting temperature (*T*_m_) and cold crystallization temperature (*T*_cc_) were determined from the DSC second heating cycle curve. *T*_g_ has been determined from the inflection point of endset and onset on DSC curve and both *T*_cc_ and *T*_m_ have been determined for the peak of the curve. Additionally, the degree of crystallinity (*X_c_*%) was evaluated according to Equation 1:

(1) $$X_{c}\left(\%\right)=\frac{∆H_{m}-∆H_{cc}}{f\times ∆H_{m}^{c}}\times 100,$$

where $∆H_{m}$ is the melting enthalpy and $∆H_{cc}$ is the cold crystallization enthalpy of each formulation. $∆H_{m}^{c}$ is the calculated melting enthalpy of purely crystalline PLA, being 93 J/g according to [19].

Dynamic thermo-mechanical analysis (DMTA) was performed in rectangular samples sized 40 × 10 × 4 mm^3^, using a TA Instruments AR G2 rheometer (New Castle, DE, USA) from 30 to 140 °C with heating rate 2 °C/min and oscillation frequency of 1 Hz and 0.1% of maximum deformation.

For DSC, TGA and DMTA assessment, three samples were analyzed for each test and the mean and standard deviation of the values are reported. Significant differences in the properties were statistically analyzed under the same parameters stated above.

For microstructural evaluation, scanning electron microscopy (SEM) micrographs from the fracture surface of the impact specimens were obtained using a Phenon SEM equipment of FEI (Eindhoven, The Netherlands) with a voltage of 5 kV. The samples were fixed to the carrier with double-faced graphite adhesive and subsequently coated with a gold–palladium alloy to allow conductivity, using a Sputter Mod Coater Emitech SC7620, Quorum Technologies (East Sussex, UK). 

Wettability was evaluated by means of the static contact angle measuring Theta angle of a distilled water drop created on a surface of the sample, using an EasyDrop-FM140 optical goniometer from Kruss Equipments (Hamburg, Germany) equipped with a camera and Drop Shape Analysis software. The test was performed at room temperature. The color evaluation was performed on a Colorflex-Diff2 458/08 colorimeter from HunterLab (Reston, VA, USA). The test was performed using CIELab* model by determining L*, a*, and b* coefficients. The L* coefficient presents darkness of the color, a* coefficient shows the intensity of green and red colors and b* coefficient shows the intensity of blue and yellow color. For both properties, 10 different measurements for each sample were obtained and averaged. The mean and standard deviation of the values are reported, and the significant differences were statistically analyzed using the same parameters previously described.

## 3. Results and Discussion

### 3.1. Characterization of Wool Fiber Treatment

**Figure 3:**
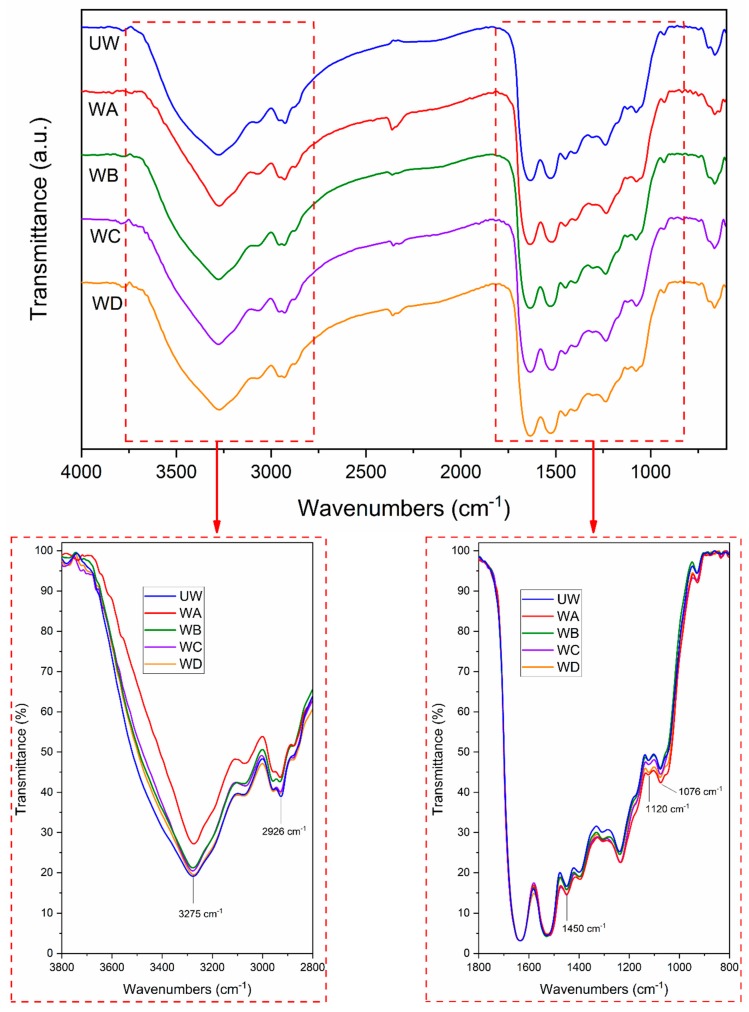
Fourier transform infrared (FTIR) spectra of unmodified wool (UW) and wool modified with studied coupling agents (WA, WB, WC, WD).

FTIR spectra of dried unmodified and modified wool fibers (with coupling agents A, B, C and D, respectively) are shown in Figure 3. All the wool samples exhibit a broad peak around 3275 cm^−1^ that corresponds to the presence of –OH group. Samples modified with coupling agents show a slight reduction of the intensity of the peak that could indicate a lower amount of –OH groups due to reactivity with the coupling agents. The biggest change of the intensity of the –OH peak was observed for wool modified with coupling agent A, producing a sharper peak. Similar change into sharper –OH peak was reported for FTIR spectrum of oxidized wool fiber modified with (3-(methacryloyloxy)propyl)-trimethoxysilane [37].

Some differences can also be observed in peaks related to –CH3 groups, resulting in peaks around 2930 and 2950 cm^−1^. These peaks are slightly different in wool samples treated with coupling agents (WA, WB, WC, WD) compared with those detected in untreated wool (UW) sample. Both peaks show a slight shift into higher wavelength values in treated samples: UW shows the peak at 2926 cm^−1^, while after wool modifications, the peaks were shifted to 2929 and 2931 cm^−1^ for WC and WB, respectively. Based on the work reported by Conzatti et al. (2014) [37], this behavior refers to the presence of small amounts of coupling agents on the wool surface, as a band corresponding to silicon and titanium compounds can be observed in the same region.

The presence of coupling agents on the fiber surface can also be explained by the increment of the peaks occurring in the range from 900 to 1400 cm^−1^. These peaks are related to the presence of –Si–O–R– and –Si–O–Si– bonds [37]. Compared to FTIR spectrum of unmodified wool (UW), the spectra after treatment show a slight increase of the intensity of the peaks in the mentioned region, especially those close to 1450 and 1100 cm^−1^, related to Si- and Ti-based coupling agents used for wool fiber modification in this study.

Finally, the changes in the intensity of peaks of FTIR spectra are slight due to the low concentration of silanes and alkoxide in the fiber treatment process (since the coupling agents’ concentration was 1 phr based on 100 parts of wool). A significant change of bands intensity was reported by Conzatti et al. (2014) [37], where coupling agent was clearly visible at 10 times higher concentration (10 wt % of coupling agent used for wool coupling). 

**Figure 4:**
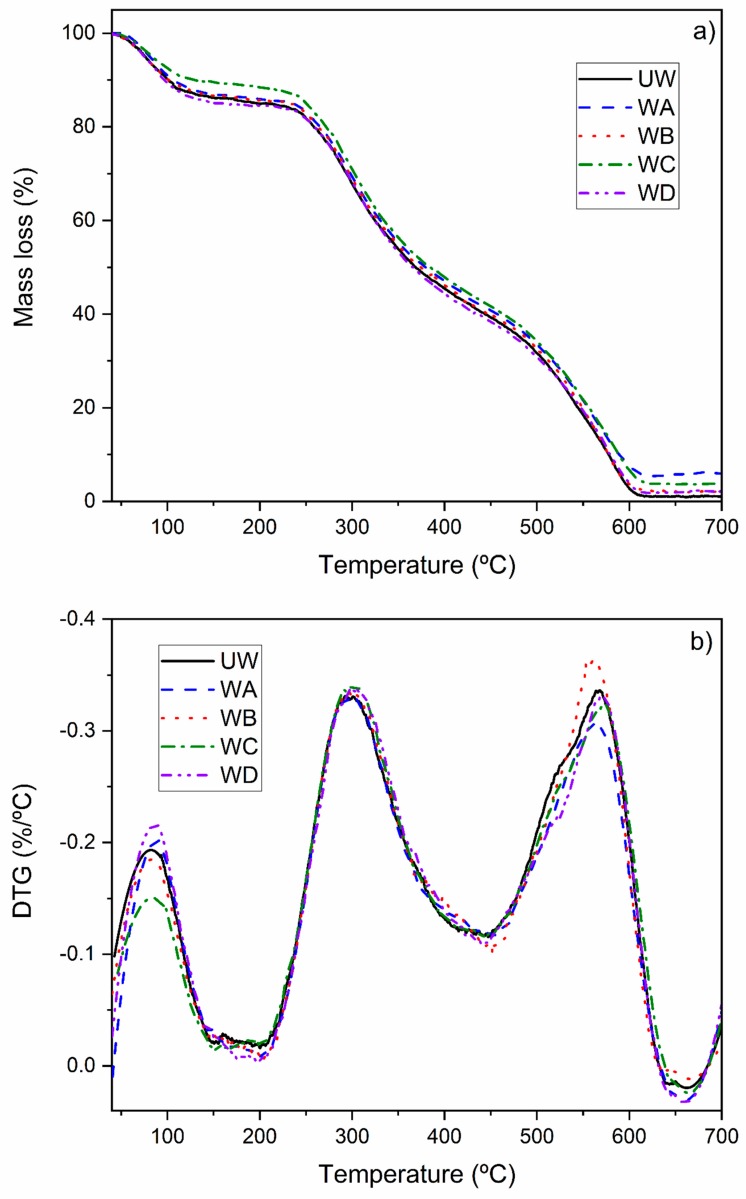
(**a**) Thermogravimetric analysis (TGA) and (**b**) first derivative of the TGA curve (DTG) of unmodified wool (UW) and wool modified with studied coupling agents (WA, WB, WC, WD).

Additionally, wool fibers before and after modifications were assessed by TGA analysis, the results of which are shown in Figure 4. The first stage of mass loss occurs at about 100 °C, which can be associated with water evaporation. Presence of water in the samples is caused by the natural water uptake tendency of the fiber. Differences in this stage between samples can be explained by different uptake of humidity on the wool surface.

Furthermore, both TGA and DTG curves do not show changes between 136 and 185 °C, which refer to the lowest boiling point among the four pure coupling agents and processing temperature, respectively. No changes in the range allow unmodified and modified wool to be used as reinforcement and to be processed under the selected conditions (180 °C) conditions. Moreover, as no significant mass loss can be observed at higher temperatures up to 250 °C, such filler can be used for other thermoplastic polymers such as polyethylene or polypropylene.

Finally, differences between unmodified wool and modified wool can be observed by the residue mass in the range between 600–700 °C. At this temperature range, the mass of all types of wool is stable, which indicates the complete decomposition of organic matter. This allows verifying how much additional inorganic matter is present in modified wool samples. The lowest amount of additional inorganic matter compared to unmodified wool was between 0.98% and 1.08% for WB and the highest amount was between 4.57% and 4.92% for WA. Such change suggests the presence of silicon and titanium-based compounds derived from coupling agents used in the work.

### 3.2. Mechanical Properties

The results of the mechanical characterization of the studied materials are shown in Table 1.

According to tensile properties results, shown in Table 1, PLA modified with plasticizer (PLA–MLO) has significantly higher resistance (*p* < 0.05) compared to other FRP, treated and unmodified wool. Nevertheless, PLA–MLO–UW presents lower resistance comparing to silane and isopropoxide treated wool materials. The Young’s modulus in all studied materials presents comparable values (values are not significantly different, *p* > 0.05), although there is a visible increase in FRP containing coupling agents A, C and D (PLA–MLO–WA, PLA–MLO–WC and PLA–MLO–WD, respectively) and decrease in the materials with unmodified wool fibers (PLA–MLO–UW) and with wool treated with coupling agent B (PLA–MLO–WB). A similar effect has been observed for epoxy resin with basalt fibers [17] or epoxy resin with jute additive [33], where surface-modified fibers showed higher tensile strength or Young’s modulus. Comparing the results in tensile properties of materials with treated wool with the PLA–MLO–UW formulation, it is possible to confirm that the fiber surface was modified, and therefore, treated wool fibers interact in a better way with the polymer matrix. The bibliography attributes this interaction to the silane or alkoxide bonds [33].

On the other hand, if the values of elongation at break are compared (Table 1), it is possible to see a very important improvement of the FRP with treated wool, provided by the coupling agents (values are significantly different, *p* < 0.05). Even though MLO plasticizer is known for its positive effect on ductile properties [19], conferring a 6.8% of elongation at break to PLA–MLO, the incorporation of 1 phr of unmodified wool results in an increase of about 15% in elongation of the material. Similar values of this property are obtained with silane-modified wool for PLA–MLO–WC. Besides, the other modified wool materials (those obtained with coupling agents A, B and D) present the highest values in elongations at break (34% on average). In addition, the standard deviation of the values of this property reveals that the wool FRP could not present much homogeneity compared to the PLA–MLO and the statistical analysis shows that there are no significant differences, *p* > 0.05, in the elongation at break of the materials that contain wool. Similar behavior and improvements in respect to elongation have been observed in PLA FRPs containing bamboo fibers after alkali surface treatment [18].

Regarding the entire tensile properties, surface treatment with each coupling agent studied (A, B, C and D) improves the wool FRP properties respect to unmodified wool material (PLA–MLO–UW) and the matrix PLA–MLO, with PLA–MLO–WD (which contains the titanium (IV) (triethanolaminate)isopropoxide) being the material that presents the highest combination of tensile properties. It shows that alkoxide coupling agent either provides a stronger bond between wool and PLA or has better reactivity than silane coupling agent.

Regarding flexural properties, results in Table 1 show that the tendency of these properties are similar to those of the tensile properties, with PLA–MLO presenting the highest flexural strength of all the studied materials. In this property, all values are significantly different. In addition, the samples containing unmodified wool (PLA–MLO–UW) exhibit lower flexural resistance than those materials that have modified wool. The highest improvement, compared to unmodified wool FRP, was notified in PLA–MLO–WB samples, which were treated with coupling agent B (trimethoxy (2-(7-oxabicyclo(4.1.0)hept-3-yl)ethyl)silane). Indeed, a positive impact on flexural strength, attributed to silane even in low concentration of the coupling agent, was observed for epoxy resin containing modified jute [33] or basalt fibers [6]. On the other hand, flexural modulus presents similar values in all the studied group of materials. No significant effect (*p* > 0.05) of wool or treatment with coupling agents was observed in the values of this property.

Charpy’s impact energy values, reported in Table 1, reveal the same behavior of tensile and flexural properties. This means the highest impact resistance of all the group of materials is observed for PLA–MLO, which is significantly different from all the wool FRP materials, except for the PLA–MLO–WB. Meanwhile, among the materials that contain wool, the highest value of this property is detected in PLA–MLO–WB. It is already known that modification of the PLA matrix with MLO is prone to increase impact resistance [19]. However, wool fibers offer a negative impact response decreasing the property by about 10 J/m^2^ comparing to PLA–MLO sample. Nevertheless, wool surface treatment with coupling agents allowed the samples to absorb more energy (in the range of 1.2 up to 6.2 J/m^2^) compared to the FRP with unmodified wool (PLA–MLO–UW). A similar effect of silane coupling on impact resistance was observed for epoxy resin with basalt fiber, although the effect between PLA and wool is significantly lower than the resin and inorganic basalt fiber [31]. Such behavior occurs because there is a better load transfer from the polymer matrix to fiber provided by the coupling agents [6].

Finally, regarding hardness values reported in Table 1, the tendency of the effect of treated and unmodified wool remains like the other mechanical properties previously discussed. In other words, there is a positive effect of coupling agent treatment on the FRP properties. In this case, PLA–MLO–WD and PLA–MLO present the highest values of hardness, which are significantly different (*p* < 0.05) from PLA–MLO–UW. Hardness results present a similar change tendency compared to maximum resistance values from the tensile test.

**Figure 5:**
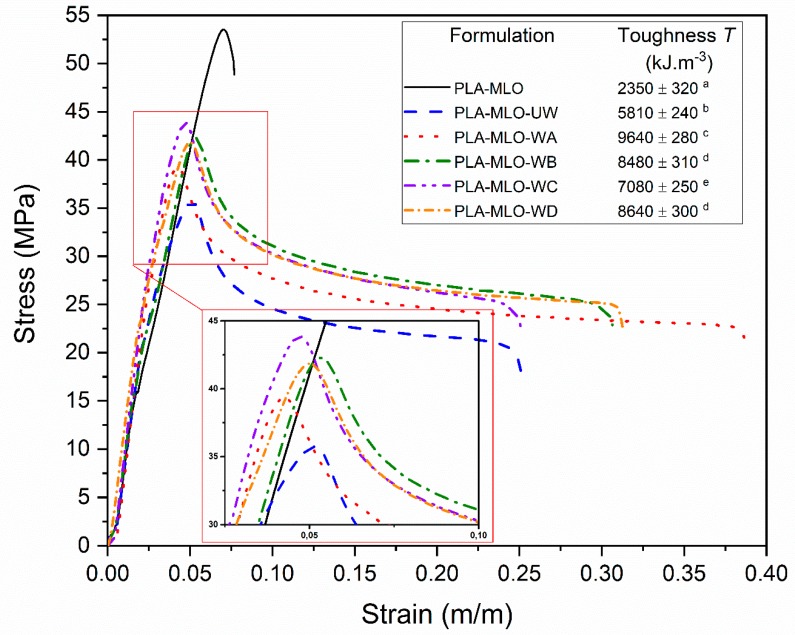
Typical stress–strain curves of PLA–wool FRP, and its corresponding toughness values. ^a–e^ Different letters show statistically significant differences between formulations (*p* < 0.05).

Figure 5 shows the typical stress–strain curves of all the formulated materials accompanied by the corresponding toughness values (*T*). It is possible to notice that the toughness of all formulations increased considerably compared to the formulation of PLA–MLO and PLA–MLO–UW. All values are significantly different since *p* < 0.05, except for PLA–MLO–WB and PLA–MLO–WD. This means that all the coupling agents studied behave in an active way in the FRP bases on PLA–MLO and wool, which confirms the idea of the fiber surface modification and the good interaction between treated wool fibers and the matrix, as previously discussed in mechanical properties. It is important to remark that even the wool fibers increase the toughness values compared to the PLA–MLO matrix. Regarding the stress–strain curves, it is possible to notice that the coupling agent A interacts better with the PLA–MLO–wool system. Therefore, the toughness of the PLA–MLO–WA is the highest of all the reported materials. Moreover, PLA–MLO–WC reports the lower values of the toughness of all treated materials. Nevertheless, its values are 200% higher than PLA–MLO and 22% higher than PLA–MLO–UW, which refers to an increase of the ductility of the materials due to the coupling agents’ modifications [36].

### 3.3. Thermal Properties

**Figure 6:**
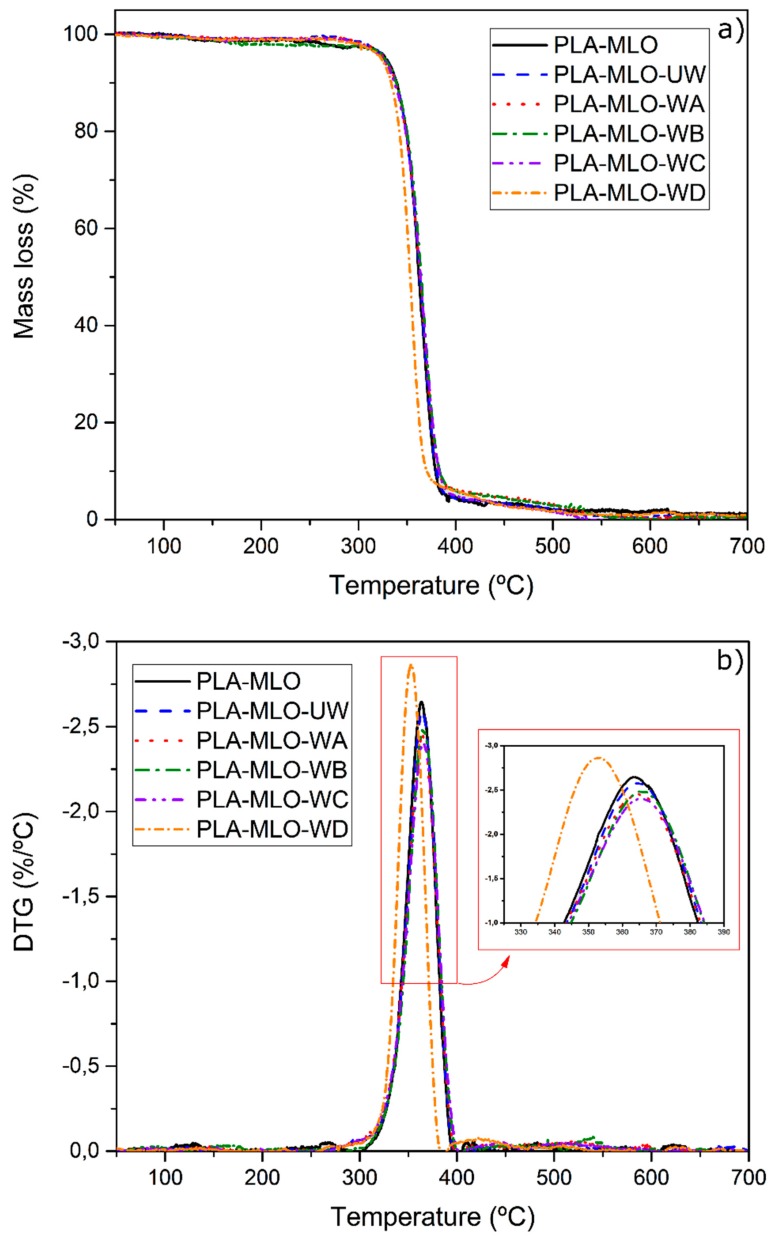
(**a**) Thermogravimetric analysis (TGA) and (**b**) first derivative of the TGA curve (DTG) of PLA–wool FRP.

Table 2 shows the values obtained from the TGA assessment and Figure 6 shows the thermogravimetric curves, for all the studied materials. All the formulated materials showed one degradation step, observed in temperatures over 300 °C. For the PLA–MLO formulation, the temperature of 5% of mass loss (*T*_5_) was estimated at 327 °C. Further modification with unmodified wool does not modify this thermal stability since no significant differences were found between the temperatures values of all samples, as shown in Table 2. Similar behavior can be found in the bibliography for PLA systems modified with nanocellulose [5] or PLA modified with nanochitin [16], and also synthetic polymer system like PVC modified with lignin [14], where no degradation under 200 °C occurred. This confirms the positive influence of bio-based additives on the thermal stability of various polymers. However, modification of wool with coupling agents tends to decrease the *T*_5_ temperature, compared to PLA–MLO–UW, inducting lower stability of the FRP, even though the values are not significantly different. The lowest *T*_5_ was observed in PLA–MLO–WC. 

Concerning to the temperature of maximum degradation (*T*_max_) reported in Table 2 and shown in Figure 6b, it is possible to observe that coupling agent D produces a reduction of the thermal stability of all formulations studied, causing a *T*_max_ about 10 °C less than the others in the group. Nevertheless, the values reported are not significantly different since *p* > 0.05. The PLA–MLO matrix, as well as all wool-containing materials, maintain the same temperature *T*_max_ between 364 and 367 °C.

Regarding the mass loss at a defined temperature (300 and 350 °C), reported in Table 2, it is possible to notice that at 300 °C the fastest material degradation was observed for PLA–MLO with the value of 2.8%. On the other hand, the lowest mass loss at this temperature was detected in PLA–MLO–UW, PLA–MLO–WA, and PLA–MLO–WD, which means that unmodified and modified wool fibers reinforced the thermal stability of the material in the PLA decomposition process. Materials treated with coupling agents B and C report similar behavior as PLA–MLO and no significant differences were found between them. At 350 °C, the mass loss of all the formulations are similar, except in the PLA–MLO–WD, which presents a statistically significant difference with the other formulations studied, with a mass loss more than 30% versus the 21–23% of the other systems. This confirms the reduction of the thermal stability in this formulation. Instead, the material modified with coupling agent B shows slightly higher stability at 350 °C, with 21% of mass loss.

**Figure 7:**
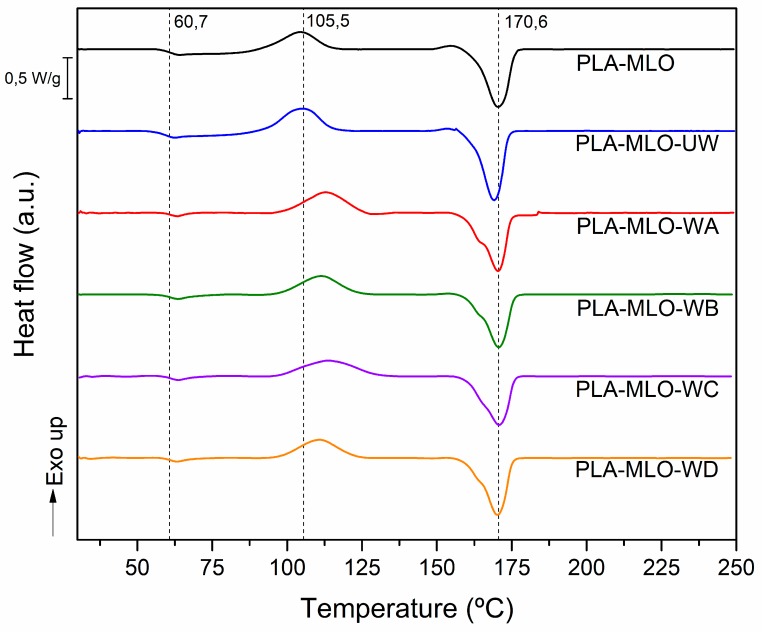
Differential scanning calorimetry (DSC) thermograms of PLA–wool FRP studied, the main transition temperatures are specified.

Concerning the DSC assessment, Table 2 shows the results of *T*_g_, *T*_cc_, *T*_m,_ and *X*_c_ for the studied materials and Figure 7 shows the DSC thermograms. It is possible to see that the wool incorporation to the FRP and the further modifications with coupling agents do not modify the PLA–MLO glass transition since *T*_g_ values remain constant in all formulations and no significant differences were found between them (*p* > 0.05). Similar behavior of glass transition temperature was observed for PLA modified with basalt or carbon fibers [38]. Nevertheless, PLA–MLO–UW tends to have the highest decrement of the *T*_g_, almost 2 °C lower compared to the matrix. Such a temperature shift may indicate a combined plasticizing effect of wool fibers and MLO in the PLA matrix since the bibliography reports that the MLO has an impact on the reduction of the glass transition temperature [19]. In addition, in the system of PLA modified with limonene, a decrease of temperature was associated with plasticization effect [2]. Regarding *T*_m_, similar behavior as *T*_g_ was observed, since the temperature values remain constant and comparable to the PLA–MLO material (around 170 °C) with no significant differences between them. However, a double melt peak is detected in formulations that contain modified wool; the change in the peak shape is due to the formation of non-perfect small crystal that melts at lower temperatures causing the formation of a small peak [39].

Regarding the cold crystallization temperature (*T*_cc_), a clear effect was found in every composite containing modified wool fibers. As seen in Figure 7 and the values in Table 2, both PLA–MLO and PLA–MLO–UW exhibit the same temperature (values are not significantly different, *p* > 0.05),. However, modification of wool fibers with the coupling agents caused an increase of the peak temperature, and values show that the increment is between 5 and 6 °C. This behavior could be attributed to the wool treatment since coupling agents provide for stronger interaction between wool and PLA. This affects the crystalline zones of the PLA matrix and makes the cold crystallization more difficult. The bibliography refers to the same effect in PLA–PHB blend, where the composites with cellulose nanocrystals were modified with acid phosphate ester of ethoxylated nonylphenol, and this increased the cold crystallization temperature [5]. In addition, the values of *X*_c_ (%) show a reduction of the crystallinity in modified wool formulations with A, B, C and D coupling agents, respectively. The values show significant differences between them, since *p* < 0.05.

### 3.4. Dynamic Thermo-Mechanical Analysis

**Figure 8:**
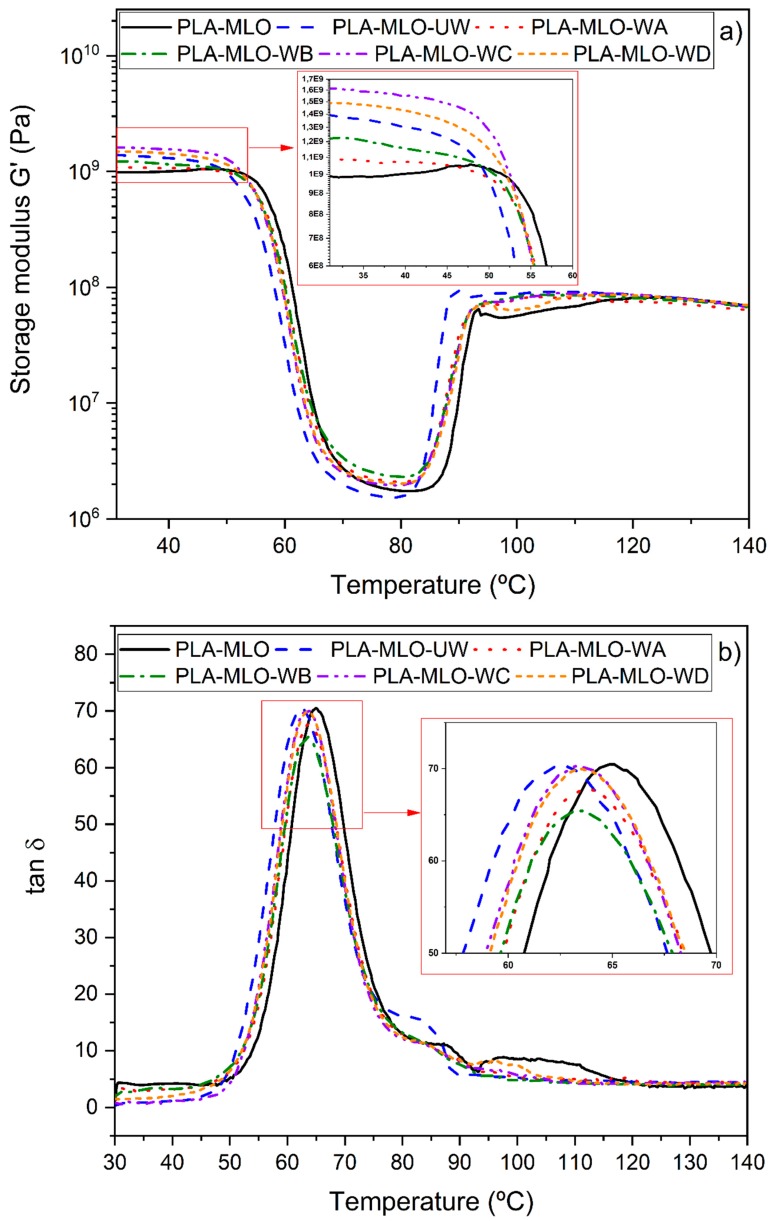
DMTA analysis curves: (**a**) storage modulus and (**b**) loss factor for PLA–wool FRP.

The thermo-mechanical behavior of the PLA–MLO–wool formulated FRP is shown in Figure 8, where the evolution of storage modulus (G’) and the gap between the loss modulus (G’’) and G’, represented by the tangent of the gap (tan δ), plotted against the temperature is presented. If the storage modulus evolution is regarded in Figure 8a, it is possible to establish that all the formulations have the same trend of these properties. Nevertheless, if the initial part of the curves is analyzed, it can be stated that the storage modulus of the PLA–MLO formulation has the lowest value of all the groups, followed by PLA–MLO–WA and PLA–MLO–WB. An improvement of the values for materials treated with coupling agents C and D respect to the PLA–MLO–UW are confirmed. This shows that more elastic behavior, up to 45 °C, can be associated with the presence of wool fibers in the FRPs. However, surface modification of wool fibers may either decrease or increase elastic behavior as compared to PLA–MLO–UW formulation. 

In Figure 8b, it is possible to see the behavior of the loss factor versus temperature of the studied FRP. In this figure, the glass transition temperature can be determined in a more exact way than DSC measurements, regarding the peak of the curves. These values are also reported in Table 3. It is possible to confirm that the trend of *T*_g_ changes is similar to the already discussed DSC results. This means that the unmodified wool incorporation to the PLA–MLO system decreases the matrix glass transition (the values show significant differences). On the contrary, the coupling agent-modified wool systems do not exhibit significant differences (*p* > 0.05) between their *T*_g_ values and those of the matrixes. The highest temperature in the range was observed for PLA–MLO, which reports a value of 64.4 °C. Modification of the blend with unmodified wool caused a decrease in the temperature of over 2 °C. This indicates an increase in polymer chain mobility due to the plasticizing effect between MLO and wool to the PLA matrix [8]. However, modification of wool fibers with coupling agents slightly increased the *T*_g_, compared to PLA–MLO–UW, and provided values in the range between 63.6 to 63.9 °C.

### 3.5. Microstructural Characterization

**Figure 9:**
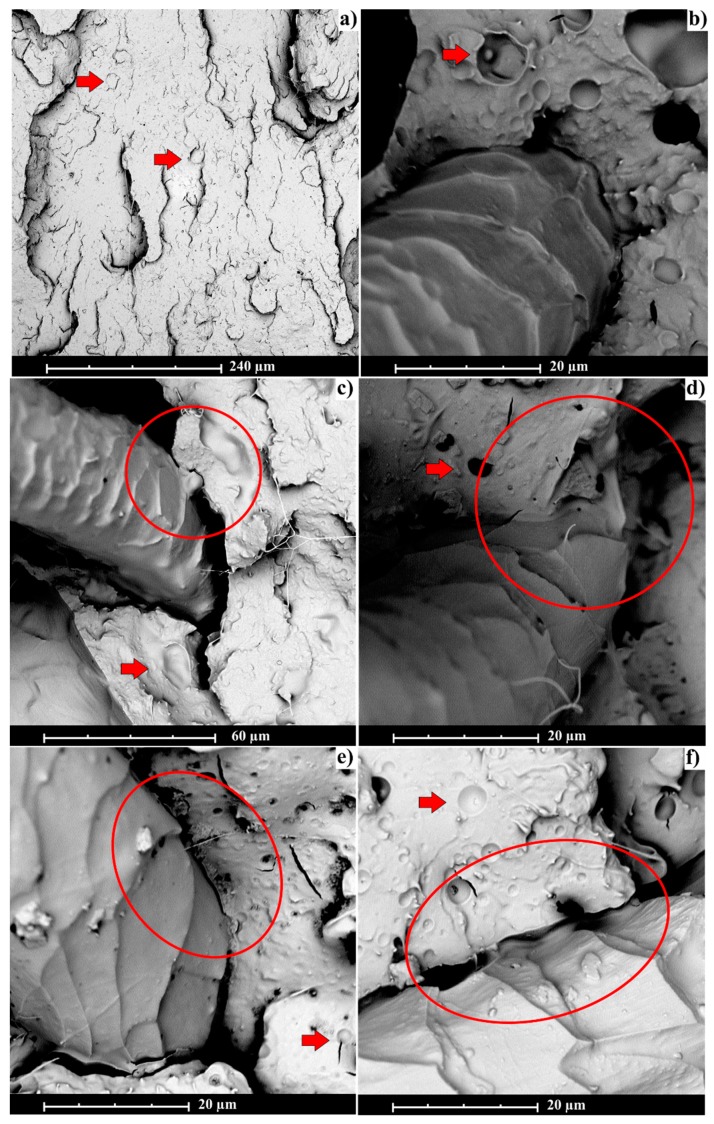
Scanning electron microscopy (SEM) images from impact fracture surface of (**a**) PLA–MLO; (**b**) PLA–MLO–UW; (**c**) PLA–MLO–WA; (**d**) PLA–MLO–WB; (**e**) PLA–MLO–WC; and (**f**) PLA–MLO–WD.

The microstructural properties of the formulate FRP were studied by SEM. Figure 9 shows the SEM images of the fractured surface for all the studied formulations. All the examined samples show good miscibility between PLA and MLO, although the PLA matrix phase still has some parts of MLO as a separate phase and can be seen in Figure 9 as sphere shapes (see red arrow) [19]. PLA–MLO (Figure 9a) has continuous surface and there is no visible fracture in the area since it is well-known that modification of PLA-based blends with MLO increases material compatibility and miscibility [8,19].

Another behavior can be noticed in FRPs that contain treated and unmodified wool. In every sample (Figure 9b–f) there is a gap between polymer matrix and wool, which is causing the failure in the mechanical performance and properties of the materials. Presence of the gap does not trigger friction during slippage [40]. In Figure 9b, weak interaction between polymer matrix and unmodified fiber is observed. Similar results were obtained for basalt fiber in epoxidized soybean oil [17] or basalt fibers in epoxidized linseed oil [6]. However, modification of the wool surface with coupling agents A, B, C and D, respectively, seems to cause an interaction with the polymer matrix (see red circles). Treated wool fibers become more rough, causing better frictional resistance according to the bibliography [40]; such effect is caused by stronger fiber surface adhesion to the surrounding polymer matrix since the surface is chemically modified [33]. In Figure 9b, corresponding to PLA–MLO–UW, the surface of the fiber is irregular and there is no evidence of interaction with PLA. In contrast, coupling agent-modified wool fibers (Figure 9c–f) show smother surface of fiber with particles and thread connected between the fiber and the matrix, which can contribute to increasing the properties of the treated wool materials compared to the unmodified one. Finally, this microstructural examination shows that the wool modification with coupling agents has a positive influence on the fiber–matrix compatibility.

### 3.6. Surface Characterization

In Table 3, the results of the color and wettability assessment is reported. Color evaluation is reported based on the CIELab* model. Results show that the incorporation of wool in the material significantly influences the brightness of the samples since L* values of PLA–MLO system are significantly different (*p* < 0.05) from the other systems. The lowest lightness has been determined for PLA–MLO formulation. Modification with unmodified wool increased the lightness by over 4 points. Although L* values of wool FRP are not significantly different (*p* > 0.05), treated wool materials increase their brightness from 43.57 (for the PLA–MLO) to 48.23 and 49.38 for formulations containing coupling agents B and D, respectively. High lightness levels in PLA-based materials were also observed when modified with limonene [2].

Referring to the a* coefficient, PLA–MLO shows slightly green color with the value of −1.89. Nevertheless, modification of such blend with unmodified wool and wool containing coupling agents A and B makes the color change into red zones, since a* increases. For PLA–MLO–WC and PLA–MLO–WD, a* has values near to 0, which means that none of the colors from red and green are dominant in those samples. The statistical analysis shows that there are significant differences between the a* values of all the formulations. 

Concerning the b* value, which refers to yellowish color zones, the lowest value of 2.38 in PLA–MLO is denoted by a slight intensity of the yellow color. Further modification of the matrix with unmodified wool increases the value to around 20 points. The values show significant differences between PLA–MLO and the formulations than contain wool, and between them, as observed in Table 3. High b* coefficient change was also observed for PLA modified with limonene, indicating a significant influence of natural filler on color properties [2]. Additionally, a change in color into brownish was observed for PLA modification with bio-origin silica–lignin filler [11].

Regarding the wettability assessment reported in Table 3, the lowest theta angle has been determined for PLA–MLO and PLA–MLO–UW, with values 66.9° and 66.4°, respectively (both values are not significantly different, *p* > 0.05). These results show the highest wettability of these formulations since PLA and wool are hydrophilic materials. Wool modifications with the studied coupling agents showed a decrease of material wettability (the material became more hydrophobic), which can be a good advantage in many applications in wet environments. The values of treated wool FRP show significant differences not only between them but also between PLA–MLO and PLA–MLO–UW. A similar increase in the hydrophobic character of materials was observed for PLA when modified with limonene [2]. Contrary to this, the surface modification of cellulose nanocrystals has increased wettability of PLA [5]. The lowest change is reported for the formulation containing coupling agent D with an average value of 75°, while the highest change has been determined for PLA–MLO–WC, with an average value of 80.8°. The results show a noticeable impact of coupling agents on surface wettability of PLA–MLO and PLA–MLO–UW.

## 4. Conclusions

Bio-based fiber-reinforced polymers (FRP) were manufactured using poly(lactic acid) (PLA) plasticized with maleinized linseed oil (MLO) and using short wool fibers as reinforcement. Composites were manufactured by extrusion–compounding followed by injection molding. Different surface treatments of wool fibers were carried out in order to improve fiber–matrix interactions. The surface modifications of the wool were confirmed by FTIR and TGA assessment. Optimum results were obtained for materials that contained wool modified with titanium (IV) (triethanolaminate)isopropoxide (PLA–MLO–WD) which presented the highest combination of tensile properties whereas the one treated with trimethoxy (2-(7-oxabicyclo(4.1.0)hept-3-yl)ethyl)silane (PLA–MLO–WB) presented the highest values of Charpy’s impact energy. The modification of the mechanical properties showed that the treatments made to the fiber have worked. Additionally, this behavior was further confirmed in the SEM analysis. 

Regarding the thermal behavior, the formulated materials showed one degradation step, observed in temperatures over 300 °C. The unmodified and coupling agent-modified wool FRP materials maintained their temperature of maximum degradation between 364 °C and 367 °C, except the PLA–MLO–WD (the sample treated with titanium (IV) (triethanolaminate)isopropoxide) that showed a reduction of the thermal stability by about 10 °C. DSC analysis revealed that the incorporation of the wool fibers modified with coupling agents to the PLA–MLO matrix caused a shift of the cold crystallization temperature to higher temperatures, and the rate of crystallinity trended to lower values. This confirmed a reduction of the crystallinity in modified wool formulations since coupling agents provide stronger interactions between wool and PLA, which makes the crystalline zone formations difficult. DMTA assessment confirmed the behavior detected in DSC testing.

As a general conclusion, we report new attractive materials from technical, economic and environmental points of view that can compete with conventional reinforced petroleum-based polyolefins such as polyethylene and polypropylene. These new proposed materials have a marked environmental efficiency as the matrix is obtained from renewable resources and the wool fiber used as reinforcement comes from the residue of the cheese and milk alimentary industry.

## Figures and Tables

**Table 1:** Tensile, flexural, hardness and impact resistance properties of poly(lactic acid) (PLA–)–wool fiber reinforced polymer (FRP).

Formulation	Tensile Properties			Flexural Properties		Impact Resistance	Hardness
	Maximum Resistance (MPa)	Young‘s Modulus (MPa)	Elongation at Break (%)	Maximum Resistance (MPa)	Flexural Modulus (MPa)	Charpy’s Impact Energy (kJ/m^2^)	Shore D
PLA–MLO	54.1 ± 1.5^a^	1816 ± 283^a^	6.8 ± 1.1^a^	27.4 ± 2.5^a^	3216 ± 131^a^	27.4 ± 2.5^a,c^	81.4 ± 2.1^a^
PLA–MLO–UW	36.5 ± 1.5^b^	1712 ± 64^a^	21.2 ± 5.1^a,b^	17.4 ± 1.9^b^	3206 ± 75^a^	17.4 ± 1.9^b^	74.2 ± 0.8^b^
PLA–MLO–WA	39.5 ± 1.3^c^	1903 ± 201^a^	36.4 ± 7.8^b^	18.6 ± 5.2^b,c^	3139 ± 132^a^	18.6 ± 5.2^b^	79.6 ± 0.5^a,c^
PLA–MLO–WB	41.6 ± 1.8^c^	1723 ± 340^a^	34.0 ± 11.5^b^	23.6 ± 5.2^c,d^	3053 ± 115^a^	23.6 ± 5.2^b,c^	77.0 ± 1.6^c^
PLA–MLO–WC	41.8 ± 1.3^c^	1923 ± 216^a^	25.2 ± 11.2^b^	20.1 ± 3.6^d^	3220 ± 25^a^	20.1 ± 3.6^b^	80.4 ± 1.3^a^
PLA–MLO–WD	40.9 ± 1.2^c^	1947 ± 129^a^	32.4 ± 9.5^b^	19.0 ± 2.0^d^	3181 ± 33^a^	19.0 ± 2.0^b^	81.0 ± 1.2^a^

^a–d^ Different letters within the same property show statistically significant differences between formulations (*p* < 0.05).

**Table 2:** Thermal properties of PLA–wool FRP studied materials.

Formulation	TGA Assessment				DSC Assessment			
	*T*_5_ (°C)	*T*_max_ (°C)	Mass Loss at 300 °C (%)	Mass Loss at 350 °C (%)	*T*_g_ (°C)	*T*_cc_ (°C)	*T*_m_ (°C)	*X*_c_ (%)
PLA–MLO	327.2 ± 0.4^a^	364.9 ± 2.5^a^	2.8 ± 0.2^a^	21.6 ± 0.6^a^	60.7 ± 0.6^a^	105.5 ± 1.2^a^	170.6 ± 0.9^a^	19.9 ± 0.9^a^
PLA–MLO–UW	327.0 ± 1.8^a^	366.4 ± 2.6^a^	1.9 ± 0.6^a,b^	23.2 ± 1.9^a^	58.6 ± 1.0^a^	105.2 ± 0.5^a^	168.1 ± 1.2^a^	19.2 ± 1.3^a^
PLA–MLO–WA	325.3 ± 1.1^a^	365.9 ± 3.3^a^	1.7 ± 0.4^b^	22.5 ± 0.9^a^	60.1 ± 1.0^a^	111.5 ± 2.0^b^	169.7 ± 0.7^a^	16.7 ± 0.7^b^
PLA–MLO–WB	326.8 ± 0.7^a^	366.9 ± 1.9^a^	2.6 ± 0.3^a,b^	21.0 ± 1.2^a^	60.2 ± 1.0^a^	111.3 ± 0.5^b^	170.1 ± 0.5^a^	16.4 ± 0.8^b^
PLA–MLO–WC	324.2 ± 1.6^a^	365.9 ± 0.3^a^	2.4 ± 0.4^a,b^	23.1 ± 1.4^a^	60.4 ± 1.4^a^	111.6 ± 2.5^b^	170.3 ± 1.4^a^	16.1 ± 0.5^b^
PLA–MLO–WD	324.9 ± 1.2^a^	356.9 ± 2.6^a^	1.7 ± 0.2^b^	32.9 ± 5.7^b^	60.2 ± 1.0^a^	110.2 ± 0.6^b^	169.8 ± 0.6^a^	17.5 ± 0.6^b^

^a–b^ Different letters within the same property show statistically significant differences between formulations (*p* < 0.05).

**Table 3:** *T*_g_ temperature determined by dynamic thermo-mechanical analysis (DMTA) from tan δ, wettability and color measurement results of PLA–wool FRP materials.

Formulation	DMTA Analysis	Wettability	Color		
	*T*_g_ from tan δ Curve (°C)	Theta Angle (°)	L*	a*	b*
PLA–MLO	64.4 ± 0.5^a^	66.9 ± 3.8^a^	43.57 ± 0.36^a^	−1.89 ± 0.05^a^	2.38 ± 0.08^a^
PLA–MLO–UW	62.3 ± 0.3^b^	66.4 ± 4.3^a^	47.88 ± 0.41^b^	1.63 ± 0.22^b^	19.98 ± 0.75^b,c^
PLA–MLO–WA	63.7 ± 0.3^a^	77.3 ± 5.8^b^	48.36 ± 0.07^b^	1.23 ± 0.15^b^	21.32 ± 0.17^b^
PLA–MLO–WB	63.9 ± 0.4^a^	76.9 ± 1.2^b^	48.23 ± 0.38^b^	0.21 ± 0.06^c^	19.54 ± 0.30^c^
PLA–MLO–WC	63.6 ± 0.4^a^	80.8 ± 2.3^c^	48.86 ± 0.33^b^	−0.03 ± 0.04^c,d^	17.21 ± 0.26^d^
PLA–MLO–WD	63.7 ± 0.2^a^	75.0 ± 0.4^d^	49.38 ± 0.45^b^	−0.36 ± 0.05^d^	18.05 ± 0.34^d^

^a–d^ Different letters within the same property show statistically significant differences between formulations (*p* < 0.05).
